# Kinetic Monte Carlo Modeling of Helium Bubble Nucleation onto Oxides in the Fe‐Ti‐Y‐O System

**DOI:** 10.1002/smsc.202400462

**Published:** 2024-12-23

**Authors:** Chris Nellis, Céline Hin

**Affiliations:** ^1^ Department of Mechanical Engineering Virginia Tech Blacksburg VA 24061 USA; ^2^ Department of Material Science and Engineering Virginia Tech Blacksburg VA 24061 USA

**Keywords:** irradiation, helium embrittlement, Monte Carlo, non-oxide dispersion-strengthened steels

## Abstract

A kinetic Monte Carlo model is developed to simulate the introduction of transmutation helium (He) atoms into nanostructured ferritic alloys (NFAs) during neutron irradiation. In this simulation, interstitial He atoms diffuse through the NFA until they become trapped within clusters consisting of other He atoms and vacancies that result from the irradiation process. The Y‐Ti‐O nano‐oxides present in the NFAs are found to be highly effective in capturing these He atoms. As a result, they prevent the formation of He bubbles at grain boundaries. Helium bubbles form on the nano‐oxides, exhibiting characteristics such as size and number density that closely resemble those observed in experimental studies. Moreover, the simulations reveal that the bubbles tend to prefer nucleation at the <111> oxide interface, and stable bubbles maintain a He‐to‐vacancy (He/Vac) ratio ranging from 1.3 to 1.8. Importantly, the presence of He bubbles is found to have a negligible impact on the segregation of solutes to the grain boundaries or on the stability of the nano‐oxides in the NFAs.

## Graphical abstract

This work describes the creation of a kinetic Monte Carlo model capable of simulating the growth of bubbles of He atoms at the interfaces of nano‐oxides in a model nanostructured ferritic alloy under neutron irradiation in anticipated reactor conditions. The model results show expected He bubble behavior and characteristics from prior experimental observations.

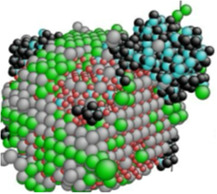

## 1 Introduction

Cladding materials within a nuclear reactor are expected to withstand the influx of helium (He) atoms resulting from (n,α) nuclear reactions occurring in the reactor. A primary mechanism leading to the degradation of metals in a nuclear reactor is the formation of large He bubbles at grain boundaries. These bubbles subsequently evolve into voids, which diminish the material's yield strength and, consequently, shorten its lifespan. This vulnerability must be addressed, especially with the next generation of nuclear reactors anticipated to operate in more intense irradiation environments with higher helium insertion rates.

Nanostructured ferritic alloys (NFAs) are a proposed class of materials designed to mitigate the degradation caused by transmutation He. The objective is to enhance resistance to He void swelling, which occurs when He bubbles reach a critical size, trapping free vacancies and expanding into voids that embrittle the material. NFAs are characterized by a high number density of very small oxide nanoparticles (typically <2 nm in diameter), typically containing Y‐Ti‐O atoms embedded in the ferritic matrix.^[^ ^1^ ^]^ These nanoparticles serve as alternative nucleation sites for He bubbles, diverting them from other defects like grain boundaries. This results in a higher density of small He bubbles within the grains, which causes less degradation of material properties than the formation of large bubbles at grain boundaries. The oxide nanoparticles in NFAs enhance irradiation resistance in two ways: 1) by keeping He bubbles away from grain boundaries; and 2) by minimizing bubble size, thereby delaying void formation. Notably, these nano‐oxides are highly resistant to dissolution at high operating temperatures^[^ ^2^ ^]^ and exhibit resilience to irradiation‐induced dissolution, maintaining their He‐capturing properties in the reactor environment.

Additionally, nano‐oxides offer other advantages such as a pinning effect that restricts dislocation movement, thereby improving the material's mechanical strength.^[^ ^3^ ^]^ The smaller grain size increases the grain boundary surface area, reducing the accumulation of vacancies by providing more opportunities for defect sinks to annihilate these vacancies.^[^ ^4^ ^]^ The oxide particles themselves act as defect sinks, further reducing the population of defects.

Research into the ability of nanostructured ferritic alloys (NFAs) to retain helium (He) atoms within their oxides is ongoing. Experimentally, it can be challenging to replicate the insertion of transmutation helium into metals outside of an actual nuclear reactor.^[^ ^5^ ^]^ To physically evaluate helium bubble distributions, helium implantation techniques have been developed to simulate reactor irradiation conditions by inducing (n,α) reactions with spallation neutrons in a surrounding blanket material, releasing He atoms into the sample while carefully controlling the He‐to‐dpa (displacements per atom) ratios.^[^ ^5^ ^]^ In some cases, accelerated ions are used instead of neutrons, although their applicability to neutron irradiation is debated.^[^ ^6^ ^]^

Studies by Kurtz^[^ ^5^ ^]^ and Yamamoto^[^ ^7^ ^]^ have investigated He bubble characteristics in non‐oxide dispersion‐strengthened (ODS) steel and the NFA MA957, respectively. These studies confirmed that NFAs reduce the size of helium bubbles and trap them at oxide sites.^[^ ^5^, ^7^ ^]^ Research by Edmondson^[^ ^8^ ^]^ on the 14YWT NFA found that ≈20% of He bubbles are located in the matrix, 49% at nanoclusters, and 14.4% at grain boundaries. When helium bubbles do nucleate in NFAs like the 14YWT alloy, they tend to be smaller in size and volume compared to conventional steel.^[^ ^9^ ^]^ Other experiments have observed that helium bubbles tend to nucleate at the <111> interfaces of oxides due to the high interface energy associated with these interfaces.^[^ ^10^ ^]^ The study notes that imaging at the 2 nm scale can be challenging, making computer modeling of oxide shapes and helium bubble locations a valuable tool.

Computer models have gained significance in nuclear materials research due to difficulties in conducting reactor experiments and the need for a comprehensive understanding of all influencing factors. When used alongside experiments, computer models enable researchers to gain insights into precise material behavior mechanisms.^[^ ^11^ ^]^

Kinetic Monte Carlo (KMC) simulations have been widely utilized in materials science to investigate processes such as precipitation during heat treatment^[^ ^12^, ^13^ ^]^ and the impact of irradiation on microstructure.^[^ ^14^ ^]^ While these models are typically developed for binary or tertiary alloys containing substitutional elements, some models also incorporate interstitial elements, as demonstrated in the Fe‐Nb‐C,^[^ ^15^ ^]^ Fe‐Ti‐O,^[^ ^16^ ^]^ and Fe‐Y‐O 9 systems. There have been previous studies involving KMC simulations with interstitial elements, including helium, in pure bcc Fe.^[^ ^17^, ^18^, ^19^ ^]^ The KMC model presented in this study is an extension of a previously developed model used to explore the precipitation and resistance of Y‐Ti‐O oxides to dissolution.^[^ ^20^, ^21^ ^]^

In this investigation, the existing KMC model for neutron irradiation of nanostructured ferritic alloys (NFAs) within an Fe‐Y‐Ti‐O system was expanded to incorporate the insertion of interstitial helium during neutron bombardment. The study focused on analyzing the size, location, and He/Vac ratio of helium bubbles within the model NFA, with comparisons drawn to experimental results. Additionally, a preliminary exploration was conducted to assess the influence of temperature on the characteristics of the helium bubbles. Furthermore, investigations delved into the potential effects of these helium bubbles on oxide stability and the segregation of solute elements within the material.

## 2 Experimental Section

### 2.1 KMC Model

**Figure 1:**
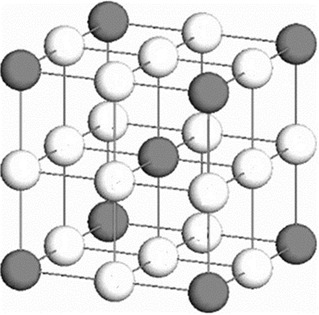
The lattice of the Fe‐Ti‐Y‐O alloy. The dark circle represents the bcc lattice site for Fe, Y, and Ti atoms and the white circles represent octahedral lattice sites for the O and He atoms.

The KMC model built upon a previous model^[^ ^21^ ^]^ utilized to investigate neutron irradiation in NFAs, incorporating the introduction of He atoms. In this framework, a fixed lattice representing the body‐centered cubic (bcc) cell was established to simulate the bcc material system. The simulation box was populated with atoms residing in substitutional sites (Fe, Ti, Y) and octahedral sites (O and He) within the bcc system. The lattice in **Figure** **1** was structured as an arrangement of eight simple cubic unit cells in a 2 × 2 × 2 configuration. Therefore, the system was analyzed from the perspective of the simple cubic framework when discussing the thermodynamic parameters.

It's important to note that the simulation box operates as a rigid lattice system that disallows elastic deformation from the perfect lattice, and is unable to generate a new phase system within the matrix. The small oxide particles within NFAs were expected to remain sufficiently small enough to preclude the formation of an independent phase, instead remaining coherent with the bcc system.^[^ ^22^ ^]^ The migration of substitutional atoms (Fe, Ti, Y) occurred through exchange mechanisms involving vacancy and interstitial dumbbell point defects. In contrast, He and O atoms migrated via an interstitial mechanism, allowing them to hop between vacant interstitial sites. During each Monte Carlo step, an event took place within the simulation box, involving either defect production or atomic migration

(1) $$\Gamma_{x}=v_{x}\times \text{exp}\left(\frac{-E_{\text{mig}}}{k_{b}}\right)$$

For each atom migration event, a jump frequency $\Gamma_{x}$ was determined, as calculated in Equation (1), taking into account the migration energy barrier $E_{\text{mig}}$ and the attempt frequency $v_{x}$, which was associated with the diffusion characteristics of each element within the bcc Fe. The energy barrier $E_{\text{mig}}$ is computed based on the specific atomic arrangement in the vicinity of the migrating atom

(2) $$E_{\text{mig}}=e^{\text{SP}}-\sum_{j}\varepsilon_{\text{Fej}}^{3}N_{j}^{3}-\sum_{j}\varepsilon_{\text{Fej}}^{4}N_{j}^{4}-\sum_{n=1,2}\varepsilon_{\text{FeO}}^{n}N_{O}^{n}-\sum_{j}\varepsilon_{jV}^{3}N_{j}^{3}$$

The local configuration was characterized by pair‐interaction energies $\varepsilon_{\text{AB}}^{n}$, where each pair interaction corresponded to the interaction between atoms A and B at the nth nearest neighbor distance in the simple cubic system. The model could encompass distances up to the 4th nearest neighbor in the simple cubic system (or 2nd nearest neighbor in the bcc system). In the model, interactions between all elements and the octahedral elements (e.g., Fe‐O, Y‐He) occurred at the 1st and 2nd nearest neighbor distances, while interactions between elements on the substitutional lattice (e.g., Fe‐Fe, Ti‐Y) occurred at the 3rd and 4th nearest neighbor distances.

The saddle point energy $e^{\text{SP}}$ was adjusted so the resulting $E_{\text{mig}}$ in pure bcc Fe aligned with the recorded migration barrier for the specific atomic species within pure bcc Fe

(3) $$\Gamma_{\text{Tot}}=\sum \Gamma_{x}$$

During every Monte Carlo step, all event frequencies were gathered to calculate the total frequency $\Gamma_{\text{Tot}}$, as shown in Equation (3). An event was then randomly chosen based on the jump frequencies $\Gamma_{x}$. The selection was made by accumulating the frequencies in the event list until the condition $\sum_{i=0}^{n}\Gamma_{x}<r\times \Gamma_{\text{Tot}}<\sum_{i=0}^{n+1}\Gamma_{x}$ was satisfied, where *r* represents a random number ranging from 0 to 1. Subsequently, the event at index *n* was executed. Following each step, the jump frequencies were recalculated, and this cycle continued until a stop condition was reached. In this model, the stop condition was met when the total radiation dose of the system, measured in displacements per atom (dpa), reached a user‐defined threshold

Certain events within the system did not have assigned frequencies and instead occurred immediately upon meeting specific conditions. For instance, the recombination and annihilation of free vacancies and interstitial dumbbells at grain boundaries happened instantaneously, following the description outlined in Soisson.^[^ ^14^ ^]^ It's important to note that when a vacancy has a helium atom as its first‐nearest neighbor, it is exempt from automatic recombination when an interstitial dumbbell is in close proximity.

### 2.2 He Insertion

In contrast to the in situ helium implantation techniques, the introduction of helium atoms into the simulation box did not involve an energy input capable of displacing atoms. All displacements in the system were a result of displacement cascades triggered by neutron impacts, with detailed information on these mechanisms provided in a companion paper.^[^ ^21^ ^]^ The rate of helium implantation was linked to the anticipated helium appm/dpa (atoms per million per displacements per atom) ratio expected from reactor conditions. Once the simulation reached a specified dpa level, a single helium atom was automatically introduced to a random location within the simulation box. This process occurred seamlessly, and as such, no event frequency needed to be established.

The literature suggested an expected helium appm/dpa ratio of ≈10 He appm/dpa.^[^ ^7^ ^]^ However, in this model, the He/dpa rate was set higher at 50 He appm/dpa to facilitate the timely formation of helium bubbles and to align with rates observed in some experimental studies.

The interactions between helium and vacancies within the KMC model were anticipated to be the most significant parameters in the helium simulations. This was because helium “bubbles” essentially consisted of large helium‐vacancy complexes where helium atoms occupied the voided spaces left by vacancy clusters. The diffusion properties of these He‐Vac complexes were detailed in a paper by Ortiz.^[^ ^23^ ^]^ While this model treated the migration of helium and vacancies within clusters as separate entities, they were expected to behave in a similar manner. In contrast to other KMC models, the clusters were not treated as distinct entities with their own migration properties. Furthermore, the association and dissociation events of individual helium atoms with clusters were not assigned separate event frequencies with specifically calculated dissociation energies. Instead, the local environment was considered through the calculation of the migration energy of vacancies using pair‐interaction energies.

### 2.3 Parameterization

#### 2.3.1 Pair‐Interaction Energies

**Table** **1** presents the pair‐interaction energies utilized in the KMC model. The rationale behind the nonhelium interactions was discussed in the companion paper, specifically concerning the nucleation of oxides.^[^ ^20^ ^]^ As there wasn't a precise estimation available for helium solubility in bcc Fe, the pair‐interaction energies for helium‐helium interactions were derived from the binding energies of two helium atoms occupying octahedral sites within pure bcc Fe.^[^ ^24^ ^]^

**Table 1:** Pair‐interaction energies for Fe‐Ti‐Y‐O system (eV) as a function of nearest neighbors.

Pair interactions	1 [eV]	2 [eV]	3 [eV]	4 [eV]
Fe‐Fe	–	–	−0.611	−0.611
Fe‐Y	–	–	−0.59	−0.52
Y‐Y	–	–	−0.57	−0.69
Fe‐Ti	–	–	−0.65	−0.53
Ti‐Y	–	–	−0.71	−0.68
Ti‐Ti	–	–	−0.69	−0.70
Fe‐Vac	–	–	−0.21	0.0
Y‐Vac	–	–	−0.35	0.0
Ti‐Vac	–	–	−0.35	0.0
Fe‐I	–	–	−0.10	0.0
Y‐I	–	–	0.25	0.0
Ti‐I	–	–	−0.10	0.0
Fe‐O	0.00	0.00	–	–
Y‐O	0.01	−0.11	–	–
Ti‐O	−0.04	−0.04	–	–
O‐O	0.10	−0.116	0.10	−0.116
He‐O	−0.34	–	–	–
He‐Vac	−2.1	–	–	–
Fe‐He	0.0	–	–	–
Y‐He	−0.46	–	–	–
Ti‐He	−0.14	–	–	–
He‐He	−0.35	−0.42	–	–

The binding energies for helium in conjunction with titanium and yttrium solutes were detailed in a paper by Vallinayagam,^[^ ^25^ ^]^ which were −0.14 and −0.46 eV, respectively. Additionally, the binding energy for helium with oxygen was recorded as −0.34 eV. These values were employed to construct the pair‐interaction energies $\varepsilon_{\text{YHe}}^{3}$,$\;\varepsilon_{\text{TiHe}}^{3}$, and $\varepsilon_{\text{OHe}}^{1}$, under the assumption that all helium interactions could be adequately described by first‐nearest‐neighbor interactions.

The interaction energy between helium and vacancies was derived from the binding energy between a single helium atom and a vacancy, as obtained from the literature.^[^ ^26^ ^]^ It's important to note that the interaction between helium atoms and interstitials is not included in this particular model.

A grain boundary was positioned at the center plane of the simulation box, and a segregation energy was factored into the migration energy calculation whenever the helium atom resided on the grain boundary. This segregation energy was fixed at 1.3 eV, in accordance with data sourced from the literature.^[^ ^27^, ^28^ ^]^ It's important to note that, unlike the segregation energy for titanium and yttrium, the contributions from entropy and enthalpy were not assessed, and the segregation energy was considered to remain constant throughout.

#### 2.3.2 Helium Diffusion Properties

In the KMC model, helium atoms were situated on octahedral sites, similar to the placement of oxygen atoms. The diffusion of helium atoms was anticipated to be rapid due to an extremely low migration barrier of 0.06 eV. A comprehensive set of characteristics is provided in **Table** **2**.

**Table 2:** Helium diffusion characteristics.

	Pre‐exponential	Migration energy	Source
He interstitial	2.8 × 10^−8^ m^2^ s^−1^	0.064 eV	[33]

The irradiation simulations, originally conducted as part of a previous irradiation study, were now being repeated. However, in these simulations, a He injection mechanism had been introduced, adding helium atoms into the system at predefined total dose intervals. The simulations tracked and documented the ultimate locations and total quantities of helium bubbles generated. Furthermore, these simulations investigate the impact of helium bubbles on the stability of oxides and the segregation of solutes at grain boundaries.

### 2.4 Simulation Procedure

#### 2.4.1 Interface Study

In an effort to gain a comprehensive understanding of helium bubble behavior, a specific simulation approach was devised. Its primary goal was to examine the preferred sites for helium bubble nucleation within the KMC model and ensure that the observed behavior aligned with expected outcomes.

**Figure 2:**
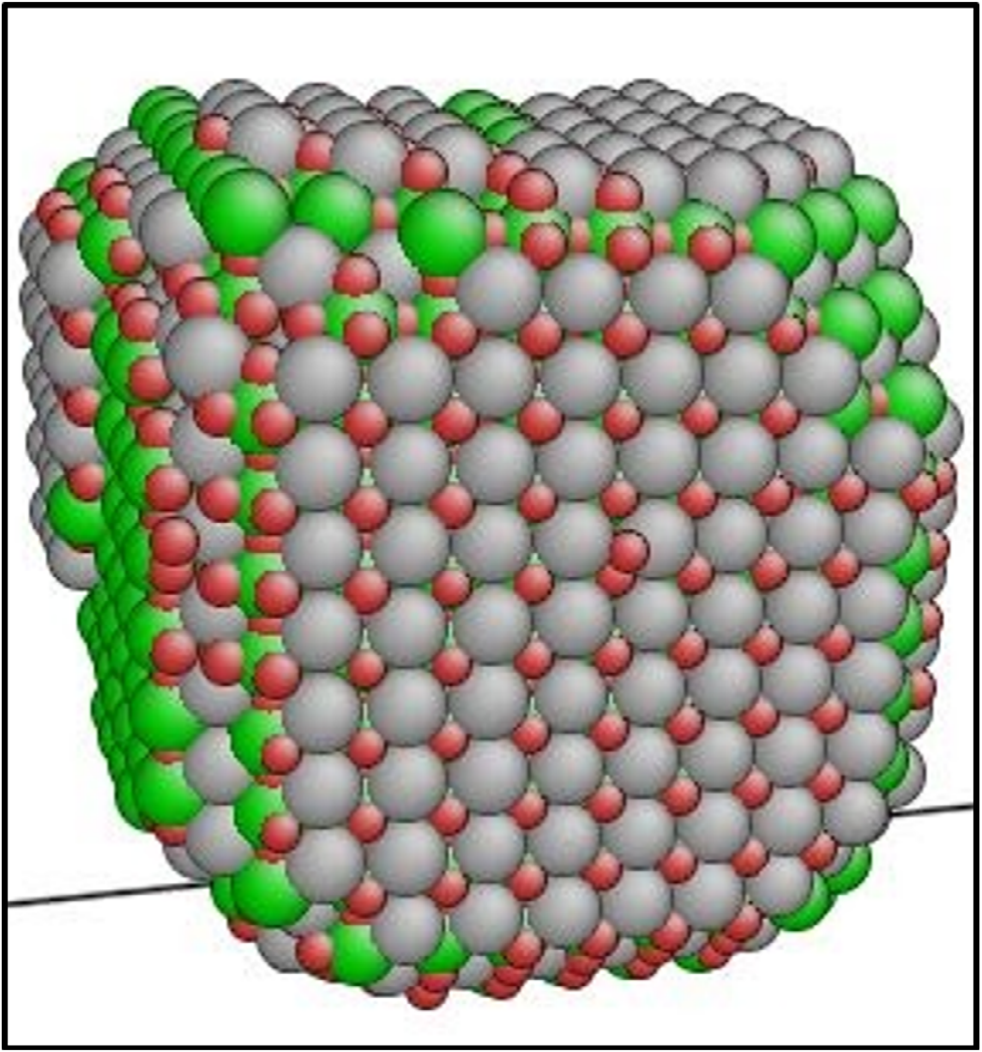
The equilibrium shape of the Y‐Ti‐O precipitate at 700 K. Green = Ti, gray = Y, and red = oxygen. Please remove the atom around the precipitates and the lines of the simulation box.

In previous KMC simulations, a substantial oxide structure was constructed in its thermally equilibrated form, exhibiting well‐defined interfaces, as depicted in **Figure** **2**. These oxides adopted a cubic shape, consistent with observations made by Ribis at 1573 K,^[^ ^29^ ^]^ featuring prominent <100> interfaces along with smaller <110> and <111> interfaces.

For the interface study, a single large precipitate, mirroring these characteristics, was introduced into a simulation box containing pure iron, along with a grain boundary. The system was then subjected to irradiation conditions specified in **Table** **3**. Since the primary focus of this particular simulation was the identification of nucleation sites, the helium appm/dpa rate was intentionally set higher than usual to expedite the growth of significant helium bubbles. After the irradiation process, the locations of the resulting bubbles were documented for future reference.

**Table 3:** Irradiation conditions for the interface study.

	Size of KMC box	Temperature	Appm/dpa	Dose rate	# Simulations
Interface Study Conditions	400 × 100 × 100 lattice points	773 K	100	$10^{-3}{\text{ dpa s}}^{-1}$	1

#### 2.4.2 14YWT Temperature Investigation

Another aspect of helium bubble behavior that was examined pertained to the impact of irradiation temperature on the size and number density of helium bubbles within the model (NFA. In this particular investigation, the material of interest was the 14YWT‐1123K alloy, utilizing the simulated atomic configuration of oxides collected from a prior study.

**Table** **4** provides a breakdown of the primary components of the nano‐oxides (Y, Ti, and O) within the iron matrix of the model NFA, designed to emulate the composition of the 14YWT alloy. It's worth noting that this model representation of the 14YWT does not encompass elements like Cr and W. **Table** **5** presents the characteristics of the oxides following isothermal heat treatment in the KMC simulation at 1123 K.

**Table 4:** The composition of the 14YWT tested.

	Y	Ti	O
14YWT atomic concentration	0.08 at%	0.27 at%	0.3 at%

**Table 5:** Oxide characteristics of the 14YWT alloy.

	Average radius	Number density
14YWT‐1123K	1.12 nm	4.7 × 10^23^ $m^{-3}$

**Table** **6** outlines the irradiation conditions that were simulated in this study. Three different temperatures were selected: 673, 773, and 873 K. A relatively high dose rate of $10^{-3}$ dpa s^−1^ was chosen due to time limitations in the timescale of the simulations. The recorded data included the average size and number density of the helium bubbles, as well as their locations (whether on oxides, within the bulk material, or at grain boundaries). To ensure comparability of helium bubble characteristics across systems with similar helium content, the stopping condition for these simulations was set to achieve the estimated appm He concentration of 400 atomic parts per million (appm) when the total dose reached 8 dpa.

**Table 6:** Irradiation conditions to observe the formation of He bubbles.

Material System	Size of KMC box	Temperature	Appm/dpa	Dose rate	# Simulations
14YWT	400 × 100 × 100 lattice points	673 K, 773 K, 873 K	50	$10^{-3}{\text{ dpa s}}^{-1}$	3
14YWT	400 × 100 × 100 lattice points	773 K	50	$10^{-5}{\text{ dpa s}}^{-1}$	3
Pure Fe (No Oxides)	400 × 100 × 100 lattice points	773	50	$10^{-3}{\text{ dpa s}}^{-1}$	3

To examine the impact of dose rate on helium bubble nucleation, the simulations for the 14YWT alloy at 773 K were repeated at a lower dose rate of $10^{-5}$ dpa s^−1^. Additionally, a simulation involving pure Fe was subjected to the same irradiation conditions at 773 K and a dose rate of $10^{-3}$ dpa s^−1^ to investigate whether the presence of oxides reduces the size of the helium bubbles. Due to computational limitations, the study of dose rate was not conducted at all tested temperatures.

Another aspect of this study was to analyze the characteristics of the helium bubbles. Stable bubbles are expected to maintain a specific ratio of helium atoms to vacancies. While this ratio has not been determined through physical experiments, energetic studies of helium bubbles have estimated the ideal He/Vac ratio.^[^ ^26^, ^30^ ^]^ Ensuring that the KMC model replicates the ideal ratio for nucleated helium bubbles would provide further validation of the model's accuracy.

#### 2.4.3 Microstructure Evolution

The presence of helium bubbles could potentially affect the long‐term stability of the oxides under neutron irradiation. Therefore, this study also examined the changes in oxide size during neutron irradiation, both with and without helium insertion. To provide a basis for comparison, a 14YWT oxide configuration was exposed to the same irradiation conditions at 773 K but without the insertion of helium. Any discrepancies in the oxide characteristics were observed and investigated. **Table** **7** outlines the irradiation conditions. Additionally, the study investigated solute segregation at the grain boundaries and assessed the influence of helium bubbles on this segregation, as radiation‐induced solute segregation at grain boundaries is a concern for the embrittlement of NFAs.

**Table 7:** Irradiation conditions to observe the formation of He bubbles.

	Size of KMC box	Temperature	Appm/dpa	Dose rate	# Simulations
14YWT conditions	400 × 100 × 100 lattice points	773 K	0.0	$10^{-3}{\text{ dpa s}}^{-1}$	3

## 3 Results

### 3.1 Interface Study

The Y‐Ti‐O precipitate was positioned within a simulation box and exposed to neutron irradiation with the He insertion mechanism activated in the KMC. The simulations were run until multiple He bubbles were detected on the surface of the oxide. A visual representation of the precipitate with He bubbles was generated using ATOMEYE software^[^ ^31^ ^]^ allowing for a closer examination of He bubble nucleation behavior.

**Figure 3:**
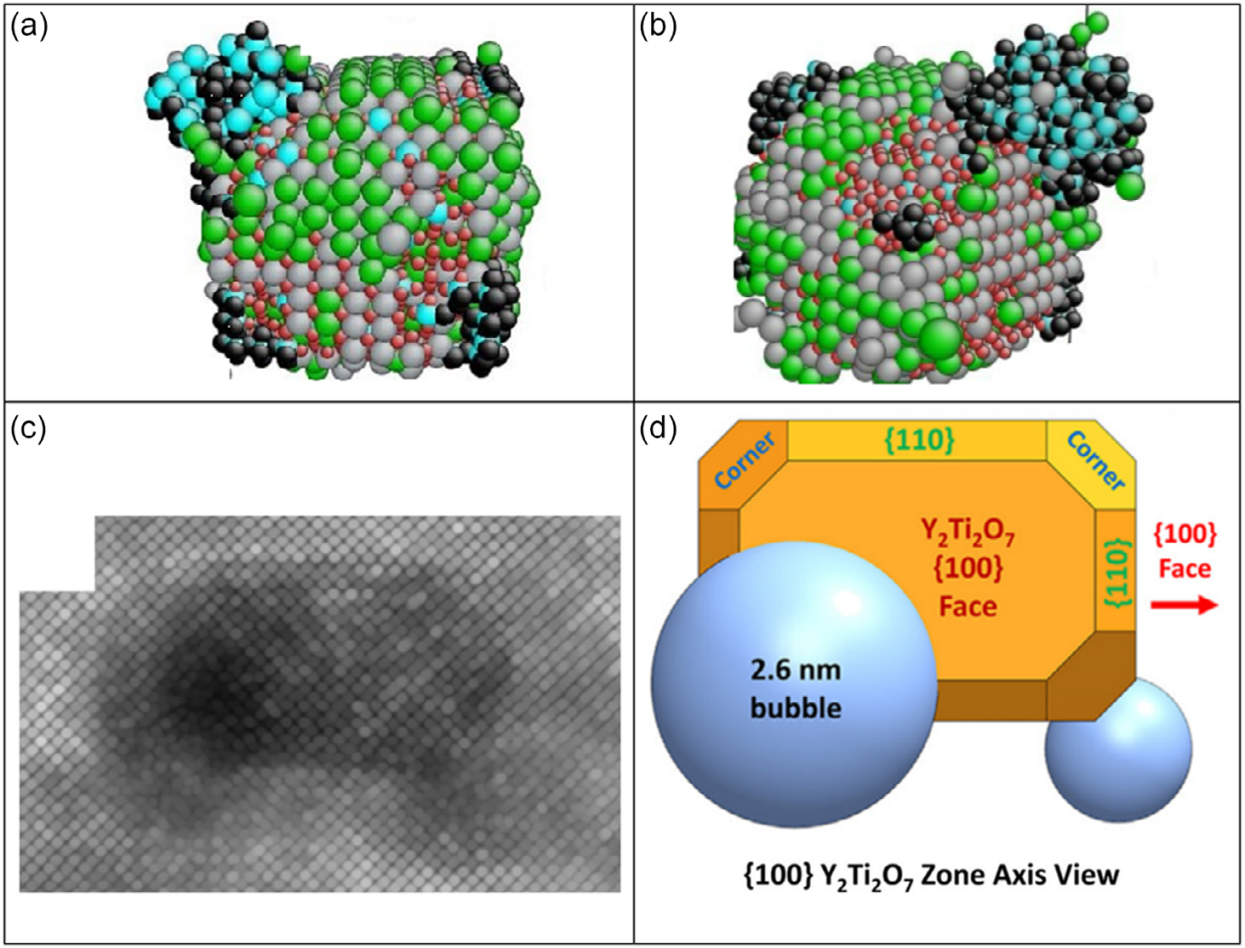
a,b) Helium bubble nucleated at the interfaces at the oxide from various angles. Green spheres: Ti atoms, gray spheres: Y atoms, red spheres: O atoms, blue spheres: vacancies, and black spheres: He atoms. c) TEM observation of He bubbles at the interface between the Y_2_Ti_2_O_7_ precipitate and the Fe matrix.^[^ ^10^ ^]^ d) Schematic of the He bubble shape, size, and location at the interface between the Y_2_Ti_2_O_7_ precipitate and the Fe matrix.^[^ ^10^ ^]^

In **Figure** **3**, multiple He bubbles are depicted, with the majority of He‐Vac complexes clustering along the perimeters of the oxide. Only the largest aggregation of He and vacancies adopts a spherical configuration. It is evident that the He bubbles exhibit a preference for nucleating at the corners of the oxide precipitates along the <111> interface. As shown in Figure 3c,d, this finding aligns very well with transmission electron microscopy (TEM) experimental observations regarding He bubble behavior in 14YWT.^[^ ^10^ ^]^

### 3.2 14YWT Temperature Investigation

Following the examination of He bubble nucleation behavior on a solitary Y‐Ti‐O oxide, the oxide distributions in the 14YWT were exposed to neutron irradiation while incorporating transmutation He. Simulations were conducted in the KMC at three temperatures, all with the same dose rate of $10^{-3}$ dpa s^−1^, continuing until the system accumulated a total irradiation dose of 8 dpa and 400 appm He. The features of the He bubbles were gathered and scrutinized for any discernible patterns or trends.

#### 3.2.1 Characteristics of He Bubbles

**Table** **8** provides data on the average size and number density of He bubbles within the 14YWT‐like alloy irradiated to 8 dpa, each containing ≈400 appm He atoms in their respective simulation boxes. A distinct pattern is evident concerning the influence of temperature: the average diameter of He bubbles increases with temperature, while the number density decreases. At temperatures of 673 and 773 K, He bubble nucleation primarily occurs at the interfaces of the nano‐oxides, discouraging nucleation near the grain boundaries. Interestingly, there is minimal discrepancy in the behavior of dose rates at 773 K. Both the $10^{-5}$ and $10^{-3}$ dpa s^−1^ cases exhibited a similar total number of nucleated He bubbles across the three simulation runs, resulting in an average size that remained consistent.

**Table 8:** Average size and number density of the He bubbles in the 14YWT after irradiation to 8 dpa.

Temperature and dose rate	Average diameter [nm]	Number density [$m^{-3}$]
673 K $10^{-3}{\text{ dpa s}}^{-1}$	0.68	$1.8\times 10^{24}$
773 K $10^{-3}{\text{ dpa s}}^{-1}$	0.81	$5.4\times 10^{23}$
773 K $10^{-5}{\text{ dpa s}}^{-1}$	0.81	$5.4\times 10^{23}$
873 K$\;10^{-3}{\text{ dpa s}}^{-1}$	1.46	$1.42\times 10^{23}$

**Table** **9** illustrates the contrast in He bubble size and density between a simulation box devoid of oxides and one containing the 14YWT composition. As anticipated, the presence of 14YWT leads to the formation of a higher quantity of smaller He bubbles in comparison to a system lacking nano‐oxides.

**Table 9:** KMC results for average size and number density of the He bubbles in the 14YWT and pure Fe after irradiation to 8 dpa at 773 K $10^{-3}$ dpa s^−1^.

Material system	Average diameter [nm]	Number density [$m^{-3}$]
14YWT	0.81	$5.4\times 10^{23}$
Pure Fe (no oxides)	1.19	$1.42\times 10^{23}$

#### 3.2.2 14YWT Experimental Replication

Alongside the simulations aimed at comprehending the temperature trends in the 14YWT NFA, we also sought to assess the KMC's capacity to replicate outcomes observed in experiments. The results obtained from the irradiation simulations conducted at 773 K were compared to those documented in Yamamoto's work.^[^ ^7^ ^]^

Experimental observations of the He bubble distribution in an NFA were observed by Yamamoto under conditions similar to our simulations at 773 K.^[^ ^7^ ^]^ The comparative analysis of these observations and the KMC results is presented in **Table** **10**. The bubble sizes closely matched, while the KMC simulation yielded nearly double the number density of He bubbles.

**Table 10:** Comparison of the He bubble characteristics between the KMC and experiment.

	Average diameter [nm]	Number density [$m^{-3}$]
From KMC	0.81	$5.4\times 10^{23}$
From Yamamoto^[^ ^7^ ^]^	0.9±0.3	$3\times 10^{23}$

#### 3.2.3 Distribution of He Bubbles

**Figure 4:**
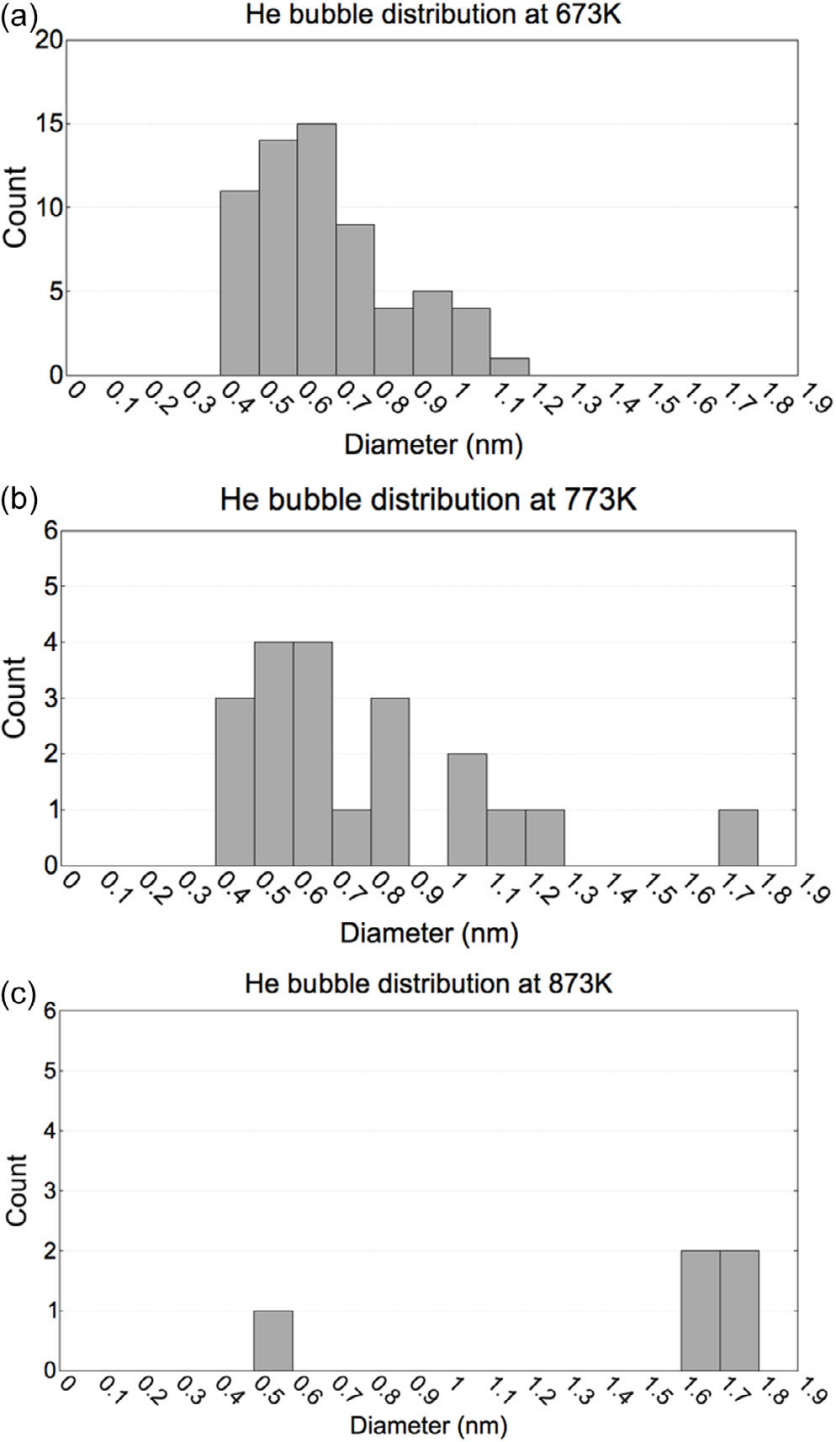
Helium bubble size distributions after irradiation to 8 dpa at a) 673 K, b) 773 K, and c) 873 K.

To gain a deeper insight into the aforementioned results, the entire distribution of He bubbles across all simulations at each temperature was gathered and visualized in the following histograms (**Figure** **4**).

Figure 4 illustrates the size distribution of He bubbles in the simulation box after 8 dpa for the three test temperatures. In the cases of 673 and 773 K, the most frequent bubbles were small, measuring less than 1 nm in diameter, while larger bubbles (>1 nm) were less common. Specifically, in the 673 K case, the bubbles were the smallest, with nearly half of them measuring less than 6 Angstroms in diameter. As the simulation temperature increased, the bubble size gradually increased, with ≈30% of them being smaller than 0.6 nm at 773 K and only one bubble being under 1 nm at 873 K. The largest bubble observed across all temperatures had a diameter of 1.8 nm.

#### 3.2.4 He/Vac Ratio

Another characteristic of the He bubbles observed was the ratio of He atoms to vacancies within these bubbles, which was compared to the ratios expected from the literature. The ratios for each bubble were collected and plotted according to their size to identify any trends in the He/Vac ratio and bubble size.

**Figure 5:**
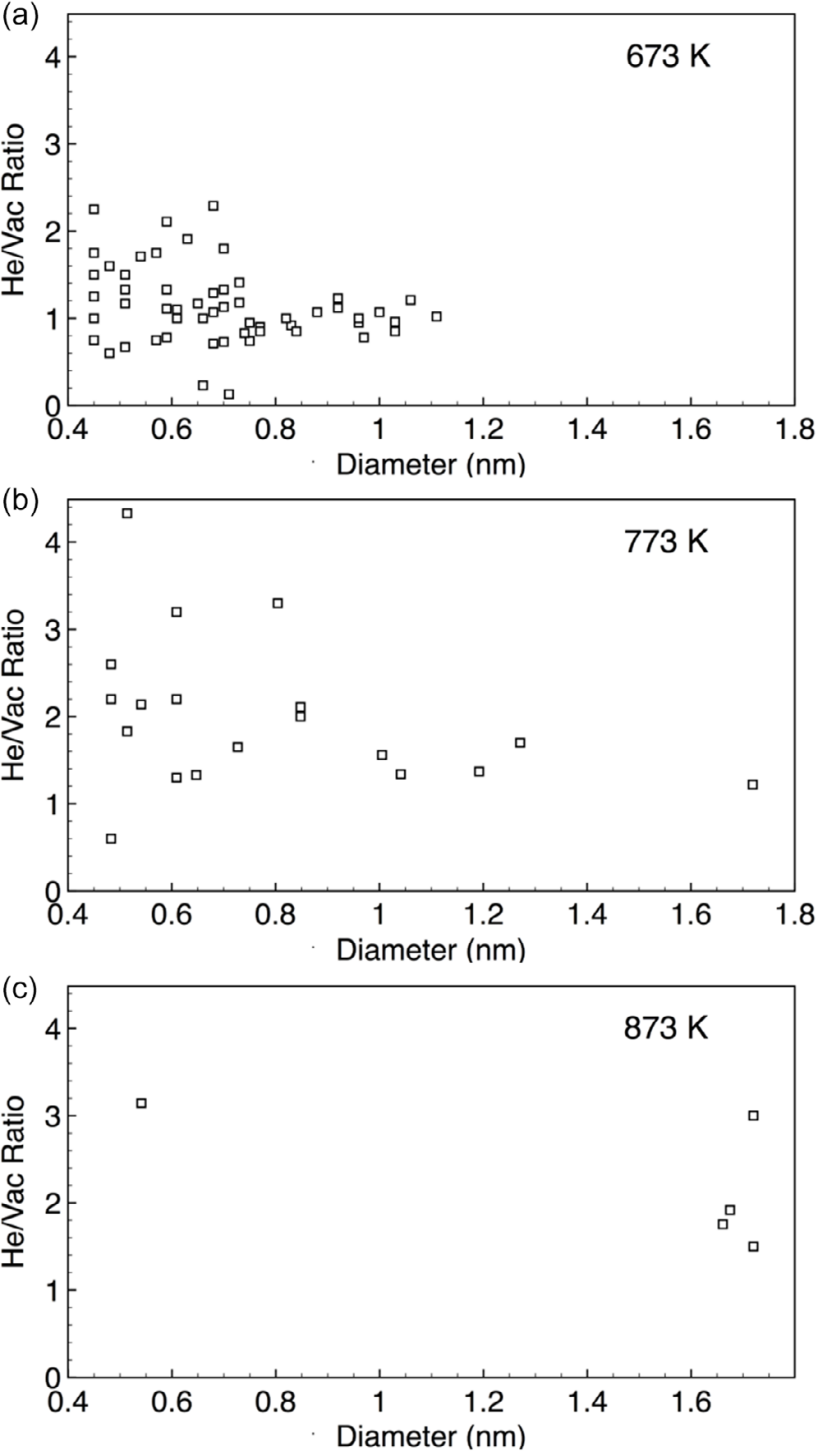
He/Vac ratio of the He bubbles in the KMC 14YWT at 8 dpa/400 appm He in irradiation temperatures a) 673 K, b) 773 K, and c) 873 K.

**Figure** **5** displays the average He/Vac ratio within the He bubbles against their size for each simulation temperature. The majority of bubbles at all temperatures exhibit a He/Vac ratio ranging between 1.3 and 1.8. Any bubbles with ratios outside this range were considered outliers. Several of these outliers were relatively small compared to the average bubble size, where a single defect or atom change could result in a substantial ratio deviation, possibly impacting their stability. Larger He bubbles, on the other hand, displayed reduced variability in the He/Vac ratio, tending to stabilize within a certain range.

### 3.3 Evolution of the He Bubble

Insights were gained into the nucleation and growth of the He bubbles. Initially, a vacancy becomes trapped at the oxide interface with the bcc Fe. Subsequently, migrating He atoms within the matrix bind with this solitary vacancy. This initial He bubble is He‐rich, but as it grows, the He/Vac ratio stabilizes within a specific range. Notably, there was no formation of voids during the irradiation simulations.

**Figure 6:**
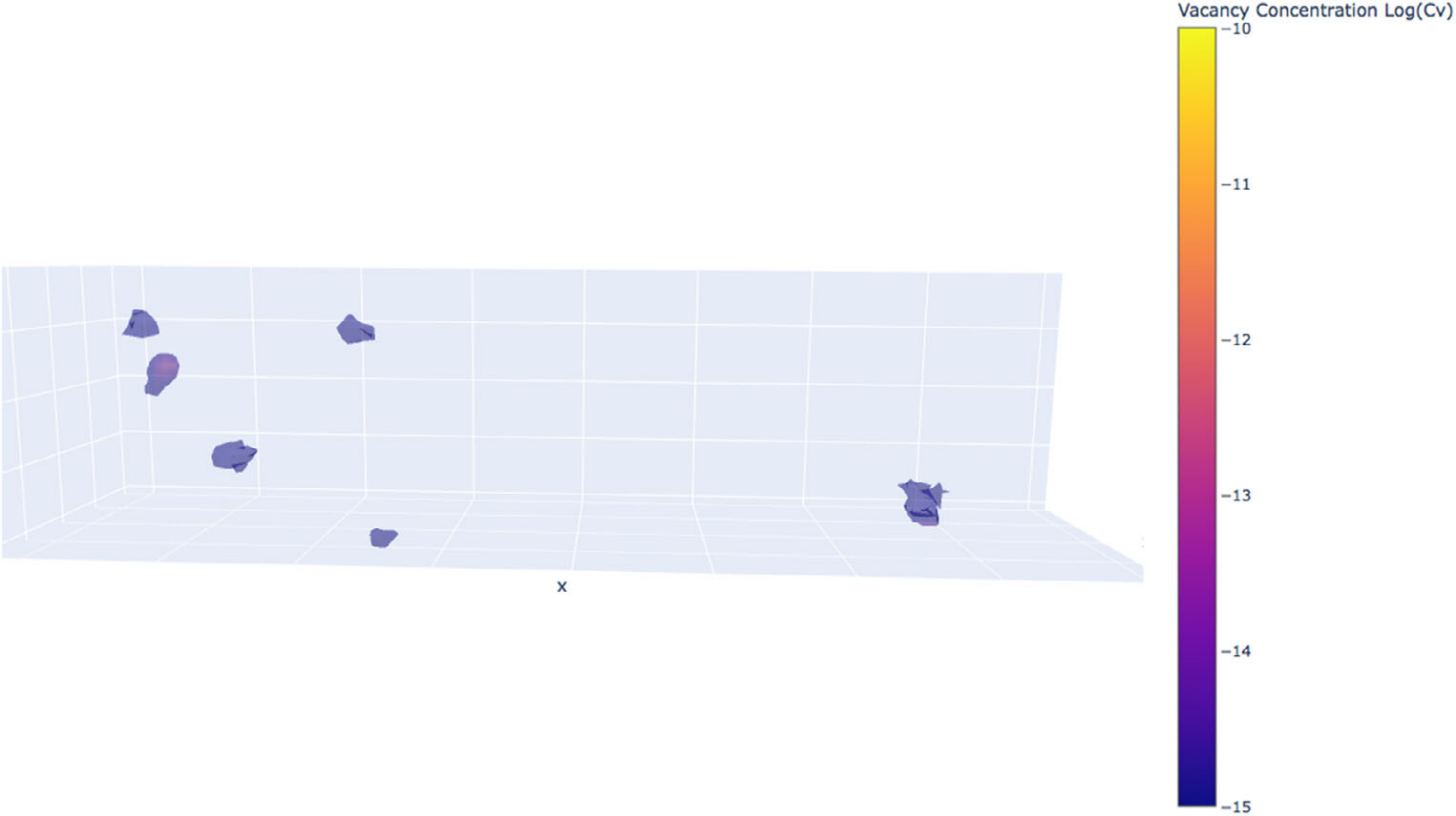
Heat map of vacancy concentration in 14YWT at 773 K irradiation simulations.

**Figure** **6** presents a heat map illustrating the movement of vacancies within the simulation box during the irradiation simulations. The regions with the highest average vacancy concentration align with the positions of the oxides. Since vacancies predominantly occupy the interfaces, it naturally increases the likelihood of He bubble formation in these areas rather than in the Fe matrix.

### 3.4 Effect of He Bubbles on Segregation and Oxide Stability

In addition to the He simulations at 773 K, a parallel irradiation simulation was conducted under identical irradiation conditions, except without the insertion of transmutation He. The concentration profile of solute atoms within the simulation box was recorded, and the segregation of these solutes in the grain boundary region was examined and compared between the two irradiation cases. Furthermore, the changes in oxide size over dpa during the irradiation treatments were investigated in both scenarios.

**Figure 7:**
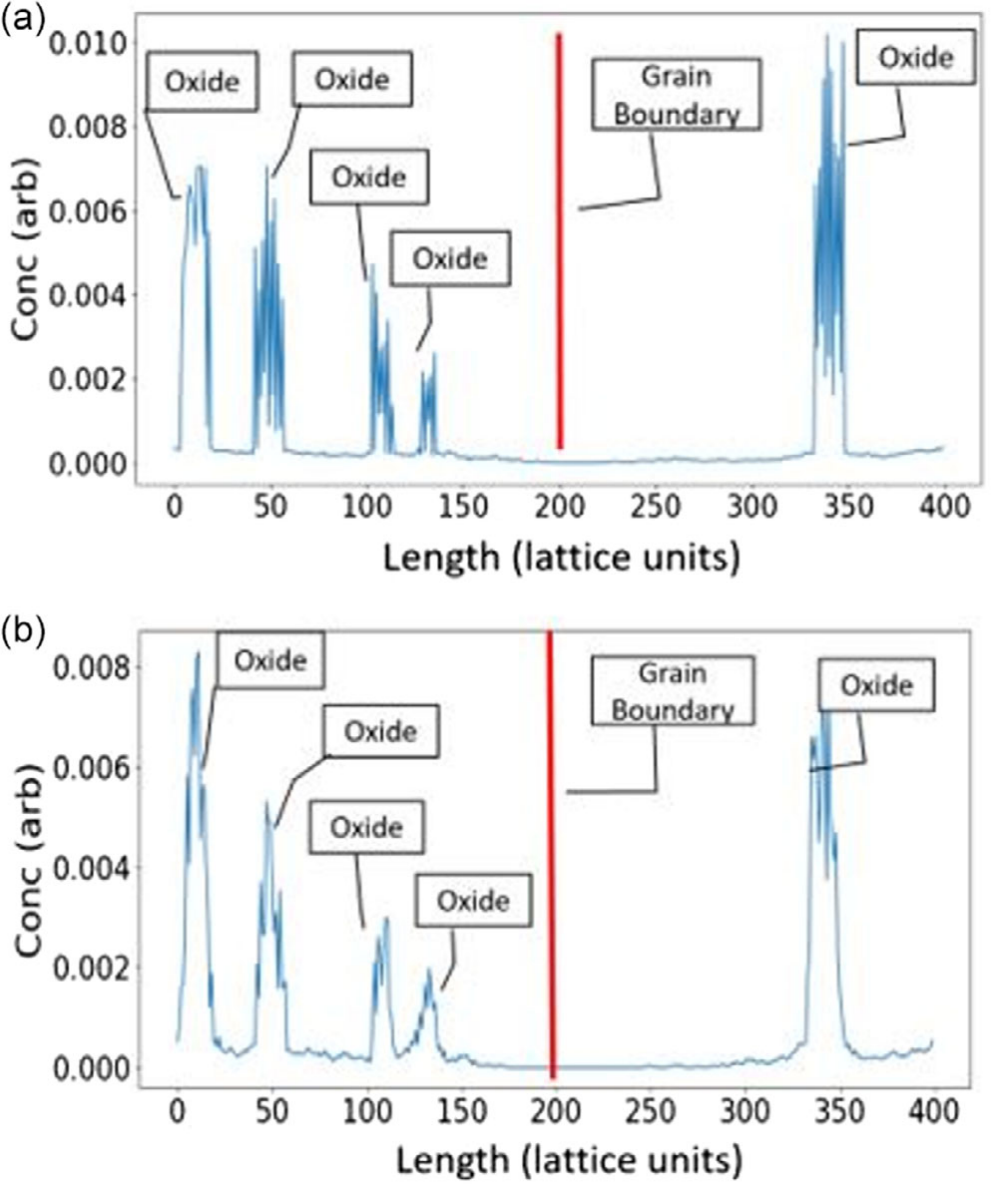
Yttrium concentration profile of the 14YWT system after irradiation to 8 dpa a) without He insertion and b) with He insertion to 400 appm He. The red line represents the grain boundary.

**Figure** **7** illustrates the concentration profiles of Y after irradiation. There is no evident enrichment of Y or Ti solute atoms at the grain boundary, whether He insertion is present or not. Instead, there is a depletion of both elements near the grain boundary. The majority of free solute atoms, not associated with an oxide, are primarily located in the vicinity surrounding the oxides.

### 3.5 Microstructure Evolution

**Figure 8:**
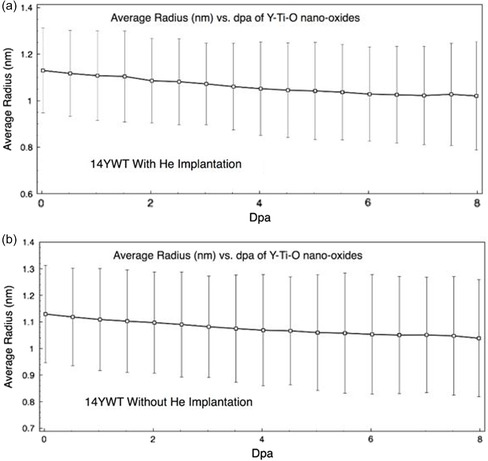
The microstructure evolution of the 14YWT at 773 K and $10^{-3}$ dpa s^−1^ a) without He insertion and b) with He insertion.

**Figure** **8** presents the microstructure evolution of the 14YWT oxides during the irradiation up to 8 dpa, with and without He insertion. In both scenarios, there is a minor reduction in the average oxide radius throughout the irradiation process, with no change in the number density and no emergence of new precipitates from the expelled solutes. Notably, there is no substantial difference in the average radius between the cases with and without He insertion.

## 4 Discussion

### 4.1 He Bubble Nucleation Behavior

The oxide particles were shown to act as alternative nucleation sites for He bubbles. Initially, these interfaces trapped vacancies which served as the initial building blocks for He bubble formation. The accumulation of vacancies around the oxides facilitated the nucleation and growth of He bubbles at these interfaces, as it is easier to join an existing bubble than to initiate a new one. This lower nucleation barrier resulted in smaller and more frequent He bubble distributions in the 14YWT alloy compared to a pure Fe sample.

The interface study conducted in Section 3.1 at the beginning of this research found that He bubbles preferentially nucleated at the <111> interfaces of the Y‐Ti‐O oxide. Even the corners without perfectly spherical He bubbles were found to be covered in He‐Vac complexes. These findings were consistent with experimental results from Stan,^[^ ^10^ ^]^ who conducted a He implantation study using larger oxides of the same composition for improved imaging. This behavior aligns with expectations from thermodynamics, as He bubbles tend to nucleate at interfaces with the highest interface energy. The <111> interface was identified as having the largest interface energy among the three primary faces of the Y‐Ti‐O oxides, as evidenced by the small surface area of the <111> interface in the shape of the oxide precipitates, since oxides reduce their surface energy by minimizing the surface area of their high‐energy interfaces.

### 4.2 Influence of Temperature

The He bubble characteristics observed at 773 K were notably different from those at 673 K, with the former having roughly 30% of the number of bubbles but twice the diameter. A similar trend was observed when comparing the 873 and 773 K simulations, where the 873 K case had half the bubble density and 50% larger diameter. This trend of a high number of small He bubbles at low temperatures and a small number of larger He bubbles at high temperatures, given the same dpa and appm He in the material, was consistent with findings in the literature. For example, investigations of He bubble characteristics in non‐NFA, non‐ODS steel (Eurofer 97) revealed substantial differences in average diameter and number density between 673 and 773 K.^[^ ^5^ ^]^ The effectiveness of the nano‐oxides in trapping He has not been experimentally observed at temperatures beyond 773 K.^[^ ^32^ ^]^

When comparing the He bubble characteristics to those found by Yamamoto,^[^ ^7^ ^]^ the KMC results showed good agreement with bubble size, though there was a discrepancy in the number density of He bubbles, although both were within the same order of magnitude. This discrepancy may be due to the limited statistics collected across the three simulation boxes. Yamamoto had more bubbles to gather statistics from, so additional simulations may be needed. The difficulty in imaging bubbles at this size may also result in an undercount.

Other factors to consider include the idealized representation of the material system in the KMC model. The NFAs have a high number of defects and dislocations within the grain that were not represented in the simulation box. Additionally, other solutes like W and Cr were not included. In a study by Kurtz,^[^ ^5^ ^]^ it was found that the preferred nucleation sites for He bubbles in a non‐NFA ferritic steel at 673 and 773 K were at the dislocations, which are still plentiful in NFAs alongside the nano‐oxides. This absence of dislocations in the simulation box could contribute to discrepancies in the results.

The He/Vac ratio of the He bubbles investigated in the KMC results aligned reasonably well with expectations from the literature. The He bubbles at 773 K provided the most well‐defined bubbles for analysis. Once the bubbles reached a certain size, the He/Vac ratio stabilized within a range of 1.3–1.8 He/Vac. Previous investigations on the stability of He bubbles, such as Morishita's^[^ ^30^ ^]^ and Fu's^[^ ^26^ ^]^ studies, found that the most stable bubbles energetically have a He/Vac ratio of 1.8 and 1.3, respectively. The observed He/Vac ratios in the KMC were consistent with these findings. The smallest bubbles with fewer than 10 vacancies exhibited the greatest variability in He/Vac ratio, mainly due to the oversized influence of their interfaces resulting from their large surface area‐to‐volume ratio.

### 4.3 Segregation

The absence of solute element segregation can be attributed to several factors. Initially, in the simulation, the majority of solute atoms were incorporated into the oxides due to their low solubility in bcc Fe at the processing temperature. The irradiation occurring at lower temperatures further reduced the solubility of these solutes in the matrix.

The presence of He bubbles in the system does not seem to significantly impact the segregation of oxide constituent atoms. Most point defects did not interact with the bubbles before being annihilated at the grain boundary. The primary mechanism driving the separation of Y and Ti atoms from the oxides was the ballistic dissolution mechanism. A free solute atom located within 1 nanometer of the oxide surface would likely remain in the vicinity, as the oxides also serve as defect sinks. All the mechanisms that apply to the grain boundary also apply to the oxide surfaces. The inverse Kirkendall effect helps keep the Y solutes close to the oxide, which is supported by the analysis of the composition change in the oxides. There was relatively little decrease in the Y component of the oxide compared to the Ti content.

### 4.4 Microstructure Evolution

The large oxides in the 14YWT alloy did not undergo dissolution under neutron irradiation with or without He insertion. However, they did experience a slight decrease in overall size, which was expected. There was no radiation‐induced precipitation of oxides in the bulk material. Similar to the segregation of solutes, the He bubbles do not seem to significantly affect the stability of the oxides. Point defects continue to migrate through the system with relatively little hindrance from the bubbles. This observation lends more support to the theory that ballistic dissolution is the primary cause of oxide shrinkage, rather than an increase in the population of point defects due to irradiation. It is possible that further irradiation to higher damage rates could have a more pronounced impact on the behavior of the bubbles.

## 5 Conclusion

A KMC model was developed to study the formation of He bubbles under typical neutron irradiation conditions in NFAs. The primary objective was to evaluate the effectiveness of Y‐Ti‐O nano‐oxides in preventing He from reaching the grain boundaries, thus enhancing resistance to radiation‐induced embrittlement.

In this model, He bubbles were observed to nucleate and grow predominantly on the Y‐Ti‐O oxides at smaller sizes and higher number densities compared to scenarios without oxides. This outcome is linked to improved resistance to radiation‐induced embrittlement in NFAs. The preferred nucleation site for He bubbles was identified as the <111> interfaces, consistent with experimental observations. The KMC model also successfully reproduced previous findings regarding bubble size and number density under different irradiation temperatures, demonstrating that bubble size increases with rising temperature. Additionally, initial investigations suggested that dose rate has minimal influence on the resulting He bubble distribution.

The next steps for the KMC the KMC model could include exploring the impact of irradiation dose rate on bubble characteristics while keeping temperature constant. Additionally, investigating the effects of varying the rate of He insertion would provide valuable insights. There is also potential for further modifications to the model, such as enlarging the simulation box size, to study the consequences of He and neutron irradiation, especially in scenarios like the 873 K case.

## Conflict of Interest

The authors declare no conflict of interest.

## Author Contributions

**Chris Nellis**: conceptualization (equal); formal analysis (lead); investigation (lead); methodology (lead); validation (lead); visualization (lead); writing—original draft (lead). **Céline Hin**: conceptualization (equal); funding acquisition (lead); project administration (lead); supervision (lead); writing—review & editing (equal).

## Data Availability

The data that support the findings of this study are available from the corresponding author upon reasonable request.
